# Editorial: Best practices for correlational research in CAPMH

**DOI:** 10.1186/s13034-023-00562-6

**Published:** 2023-02-01

**Authors:** Gerrit I. van Schalkwyk

**Affiliations:** Intermountain Healthcare, Salt Lake City, UT USA

The majority of manuscripts submitted to *Child and Adolescent Psychiatry and Mental Health* describe relationships between observed variables, either at a moment in time or longitudinally. This type of work has the potential to enhance our understanding of how specific exposures may increase risks of a certain outcome, as well as to understand variables that may mediate or moderate such relationships. Given the large number of submissions that employ this approach, the editorial team must increasingly apply a high standard during initial review. In order to provide authors with a more transparent review experience, this editorial will outline some considerations for best practices when submitting such work to our journal. These considerations will be illustrated using a case example.

1. Research needs to be hypothesis driven: it is common for manuscripts to draw on data from large datasets, and then describe statistically significant correlations between variables without a specific reason to have interrogated these variables. This represents a major limitation that will significantly reduce our interest in the submission. When authors describe a specific, well-substantiated hypothesis that guided their enquiry, the work has greater scientific validity, and will likely be of greater interest to our readers—even if the findings are statistically non-significant. For example:

*“There is considerable interest in whether early screen exposure impacts executive function. To test this hypothesis, we administered a validated measure of screen time combined with a measure of executive function to a sample of 250 youth. Our findings revealed no significant relationship between screen time and executive function”*

Would represent a more valid approach than:

*“In a sample of 250 youth, data was collected on anxiety levels, parent education, trauma exposure, and screen time. We found a statistically significant correlation between paternal high school completion and screen time exposure”*

2) The work needs to occur in the context of a theoretical framework. Beyond stating a hypothesis, high quality correlational research should be situated within existing theory, and should attempt to advance our existing understanding by asking questions that exist at the edge of our current knowledge. This will increase the impact of the work relative to research that situates its hypothesis in an arbitrary way that is entirely disconnect from the state of the field. As an example of a best practice:

*“Two main theories exist regarding how screen time impacts cognitive function. The first argues for the impact screen time has on ‘displacing’ other activities that are valuable to normal development. The second argues that screen time has a direct negative impact that is independent of displacement. The present study therefore sought to measure both screen time and time spent on other activities, such that our results could be interpreted in the context of these competing theories.”*

By contrast, a disconnected approach could be:

*“Much is unknown about how screen time impacts cognitive function. One area that is unexplored is the impact of nutrition. We therefore decided to study nutritional status and screen time and how it correlates to cognitive function”*

3) Less is more. There is a temptation when conducting correlational research to measure a large number of variables in order to increase the odds of finding a statistically significant signal. However, this approach makes the research inherently less hypothesis driven, and suggests that the researchers are less focused on *answering a question to advance our understanding* than they are on finding something that will achieve some measure of significance. When a large number of variables are tested, there are likely to be correlations identified by chance which do not represent true relationships, and may obscure rather than advance our understanding of a phenomenon. In order to reinforce more disciplined approaches to correlational research, our editorial board commits to considering negative studies that are rigorous in their design as superior to positive studies that came to significance by testing too many relationships.
4) Tell the entire story. No research is perfect, and good findings do not always come about in the context of perfectly designed studies. It is preferable that authors present an accurate and honest account of how their work was conducted, rather than to attempt to provide a tidier narrative that is not reflective of the reality of what was done. Most often, this problem will occur when a hypothesis was developed after the fact to ‘fit’ a significant finding, or when the initial hypothesis was changed for the same purpose:

*“Given the interest of the authors in the impacts of paternal education level, we sought to test the hypothesis that paternal education was correlated with excess screen time in a sample of youth”*

A more honest presentation could be:

*“We tested the hypothesis that socio-economic status would be correlated with increased screen time in a sample of youth, but this finding proved to not be significant in our sample. Of interest for further work, a secondary analysis did identify a strong relationship between paternal education and screen time exposure, which may relate to an emerging theory on how education level impacts aspects of child supervision. A future study could aim to develop and test a specific hypothesis around this possible correlation.”*

5) Make the impetus clear. Particularly for a clinically-focused journal like *CAPMH*, it is of importance that correlational work be driven by a specific clinical or theoretical problem that it is attempting to address. A significant number of manuscripts justify the topic under investigation by a vague statement of needing to ‘better understand the factors’ that drive or influence an outcome, and then conclude that ‘further research is required’, or that more resources be dedicated to addressing which ever risk factor or mediator was identified. By contrast, a more robust approach is to articulate why a specific knowledge gap is salient and important, and how the present study has the potential to provide a real improvement in our understanding that can guide specific decisions about treatment, diagnosis, resource allocation or policy. A less desirable but common approach could be described as follows:

*“Given the importance of cognitive function, it is important to understand factors by which it may be impacted. Given our finding that cognitive function is lower in youth who had one parent that did not complete high school, more resources should be given to screen these youth for cognitive problems’.*

A contrasting and superior approach would be:

*“The lack of understanding on whether screen time impacts cognitive function a) directly, or, b) by limiting other important activities, is a critical limitation in our ability to issue guidance to patients and families. Our study found that increased screen time was only correlated with reduced cognitive function in youth who also had decreased engagement in other daily activities. This supports an emerging theory that the impacts of screen time on cognitive function can be overcome by ensuring youth are also able to spend enough time on other stimulating tasks, which has both clinical and policy implications.”*

We recognize that every study is unique, and will continue to employ a review process that is individualized, and balances the strengths and weaknesses of each submission. This editorial should not be viewed as a checklist that will either guarantee or prevent submission, but a set of considerations that we hope will aid authors in the development of their work, and provide clarity on how the editorial board might evaluate a submission.

## Data Availability

N/a.

